# DNA Primase Subunit 1 Expression in Hepatocellular Carcinoma and Its Clinical Implication

**DOI:** 10.1155/2020/9689312

**Published:** 2020-08-22

**Authors:** Yipeng Zhang, Lijun Li, Renzhi Liu, Changchun Zeng

**Affiliations:** ^1^Clinical Laboratory, Shenzhen Longhua District Central Hospital, Guangdong Medical University, Shenzhen, Guangdong, China; ^2^Department of Quality Control, Shenzhen Longhua District Central Hospital, Guangdong Medical University, Shenzhen, Guangdong, China; ^3^Department of Infectious Disease, Shenzhen Longhua District Central Hospital, Guangdong Medical University, Shenzhen, Guangdong, China; ^4^Department of Medical Laboratory, Shenzhen Longhua District Central Hospital, Guangdong Medical University, Shenzhen, Guangdong, China

## Abstract

DNA Primase Subunit 1 (PRIM1) is crucial for cancer development and progression. However, there remains a lack of comprehension concerning the clinical implication of PRIM1 in HCC. Here, aberrant expression of PRIM1 was identified in HCC according to available databases. The prognostic value of PRIM1 in patients presenting with HCC was further assessed based on TCGA data. Gene set enrichment analysis (GSEA) was subsequently conducted to investigate the potential function of PRIM1. Additionally, the correlations between tumor-infiltrating immune cells (TIICs) and PRIM1 expression were evaluated. The data from TCGA, GEO, ONCOMINE, and HCCDB databases illustrated that PRIM1 was overexpressed in HCC tissues, compared to normal liver tissues (all *p* < 0.05). Kaplan-Meier analysis revealed that high PRIM1 expression in HCC was closely correlated with worse overall survival (*p* < 0.05). The univariate and multivariate analyses illustrated that PRIM1 expression was an independent novel prognostic indicator in HCC. Additionally, the area under the receiver operating characteristic (AUROC) curve for PRIM1 reached 0.8651, indicating the diagnostic significance of PRIM1 in patients with HCC. GSEA showed that PRIM1 overexpression was significantly enriched in several tumor-related signaling pathways. Besides, TIIC analysis clarified the association between PRIM1 expression and TIICs in HCC. The findings disclose that PRIM1 profoundly implicated in promoting tumorigenesis might work as a desirable biomarker for HCC.

## 1. Introduction

Hepatocellular carcinoma (HCC) with high mortality rates has become a threat to public health [1]. Although surgery is helpful for prolonging the lifetime of HCC patients, most patients have missed the opportunity for surgical treatment at the time of diagnosis [2]. Patients diagnosed with advanced liver cancer lack curative therapies and have a worse prognosis [3]. Great progress has been made in HCC treatment protocols, including radiofrequency ablation, curative resection, radioembolization, liver transplantation, and systemic targeted therapy [4–6]. Nonetheless, it remained a huge challenge to discover novel molecular biomarkers for HCC [7].

Primase, a heterodimer of two subunits, is a pivotal enzymatic element in the replication of DNA. DNA primase synthesizes small RNA primers for short DNA fragments generated throughout DNA replication [8–11]. For this reason, DNA primase, frequently referred to as RNA primase, plays an essential role in RNA polymer synthesis. Primase Subunit 1 (PRIM1), encoding a small, 49 kDa primase subunit, has the catalytic function of an enzyme. Previous studies have implied that PRIM1 closely related to osteosarcoma [12], pancreatic cancer [13], and breast cancer [14] is profoundly implicated in cancer progression [11]. However, the significance of PRIM1 in HCC remains enigmatic.

Recently, a massive number of studies have identified genes associated with the prognosis of HCC [15–18]. Until recently, there is still a lack of the accurate and effective markers for the diagnosis and prognostic of HCC [19]. Further studies are still essential to improve the accuracy in diagnosis and prognosis of HCC.

To deepen the comprehension of PRIM1 in HCC, we explored the aberrant expression of PRIM1 based on The Cancer Genome Atlas (TCGA) and the Gene Expression Omnibus (GEO) databases. Additionally, our study also explored the clinical application value of PRIM1 in HCC by Kaplan-Meier survival and Cox regression analyses. As far as we know, we illustrate the role of PRIM1 in HCC for the first time.

## 2. Material and Methods

### 2.1. Data Extraction from GEO and TCGA Databases

The RNA-Seq gene expression level 3 Htseq-count data restricted to 371 primary tumor samples and 49 normal samples were selected and downloaded from The Cancer Genome Atlas (TCGA, https://portal.gdc.cancer.gov/) [20]. In this study, we analyzed the pathological data (*n* = 377) of HCC collected from TCGA database. Ten patients with histology of fibrolamellar carcinoma (*n* = 3) and hepatocholangiocarcinoma (*n* = 7), one patient without survival data, and six patients without gene expression levels were excluded. The study comprised 360 samples of histologically confirmed HCC. Subsequently, the correlations between PRIM1 expression and clinicopathological characteristics of HCC patients were assessed.

GSE25097, GSE6764, GSE14520, GSE45436, GSE55092, and GSE60502 datasets were selected and downloaded from the Gene Expression Omnibus (GEO, https://www.ncbi.nlm.nih.gov/geo/) database [21].

### 2.2. ONCOMINE Database Analysis

The mRNA levels of PRIM1 in HCC were further elucidated based on the ONCOMINE database (https://www.oncomine.org/), which is a cancer microarray database holding a total of 86,733 samples and 715 gene expression datasets [22]. Genes were screened by fold change ≥ 2 and FDR < 0.05, and the top 10% of ranked genes were sorted and considered.

### 2.3. Integrative Molecular Database of Hepatocellular Carcinoma (HCCDB) Analysis

The Integrative Molecular Database of Hepatocellular Carcinoma (HCCDB, http://lifeome.net/database/hccdb/) database was further applied to annotate the expression patterns of PRIM1 in HCC, which is an integrated database holding a total of 3,917 samples and 15 gene expression datasets [23].

### 2.4. The Kaplan-Meier Plotter Survival Analysis

Prognostic values of PRIM1 in HCC were further evaluated using the Kaplan-Meier plotter (http://kmplot.com/analysis/) [24]. The log-rank *p* was calculated to display differential survival.

### 2.5. Gene Set Enrichment Analysis

Gene set enrichment analysis (GSEA, http://software.broadinstitute.org/gsea/) is employed to identify remarkably overrepresented or underrepresented groups of genes [25–27]. In the current study, GSEA was performed in terms of the correlation between gene sets and PRIM1 expression, and gene set permutations were carried out 1000 times. Gene sets and the correlative pathways are filtered and ordered by the nominal *p* value and normalized enrichment score (NES).

### 2.6. Tumor Immune Estimation Resource (TIMER) Database Analysis

Tumor Immune Estimation Resource (TIMER, https://cistrome.shinyapps.io/timer/) database [28] was applied to evaluate the associations between immune cells (B cells, CD4+ T cells, CD8+ T cells, neutrophils, macrophages, and dendritic cells) and PRIM1 expression.

### 2.7. Statistical Analysis

R software (v.3.6.0; The R Foundation) and GraphPad Prism version 8.0 software (GraphPad Software, Inc.) were applied for statistical analysis and scientific graphing. The correlations of PRIM1 expression and clinicopathological characteristics were evaluated by the Wilcoxon signed-rank test and logistic regression. The Cox regression and Kaplan-Meier analyses were further carried out to elucidate the association between PRIM1 expression and survival data. Furthermore, a receiver operating characteristic curve is plotted to illustrate the underlying diagnostic ability of PRIM1 in HCC. *p* < 0.05 was regarded as statistical significance.

## 3. Results

### 3.1. Patient Characteristics

Level 3 mRNA expression and clinical data from 360 primary HCC and 49 normal control tissues were obtained from TCGA database. Data on the clinicopathological features of HCC, such as sex, age, stage, body mass index (BMI), grade, alpha-fetoprotein (AFP) level, platelet, and race, was collected. As listed in Table 1, the patients, median age 60 years (range 16-90), included 243 males and 117 females. Among them, 44.3% of patients were from Asian populations, and other non-Asian populations included White (50.3%), Black or African American (4.9%), and Alaska Native or American Indian (0.6%) populations. There were 6.4% patients with <18.5 kg/m^2^ body mass index (BMI), 46.5% patients with 18.5-24.99 kg/m^2^ BMI, 26.6% patients with 25-29.99 kg/m^2^ BMI, and 20.5% patients with >30 kg/m^2^ BMI. A total of 167 patients (49.7%) had stage I disease, 81 patients (24.1%) had stage II disease, 84 patients (25.0%) had stage III disease, and only four patients had stage IV disease. Furthermore, there were 14.9% patients with G1 grade, 48.2% of patients with G2 grade, 33.8% of patients with G3 grade, and 3.1% patients with G4 grade. Moreover, this study included 23.3% of patients with AFP levels > 400 ng/ml and 76.7% of patients with AFP levels ≤ 400 ng/ml. Additionally, the median platelet was 24.1 × 10^3^/mm^3^ (range 0.004‐499 × 10^3^/mm^3^).

### 3.2. The mRNA Level of PRIM1 Is Upregulated in HCC

**Figure 1:**
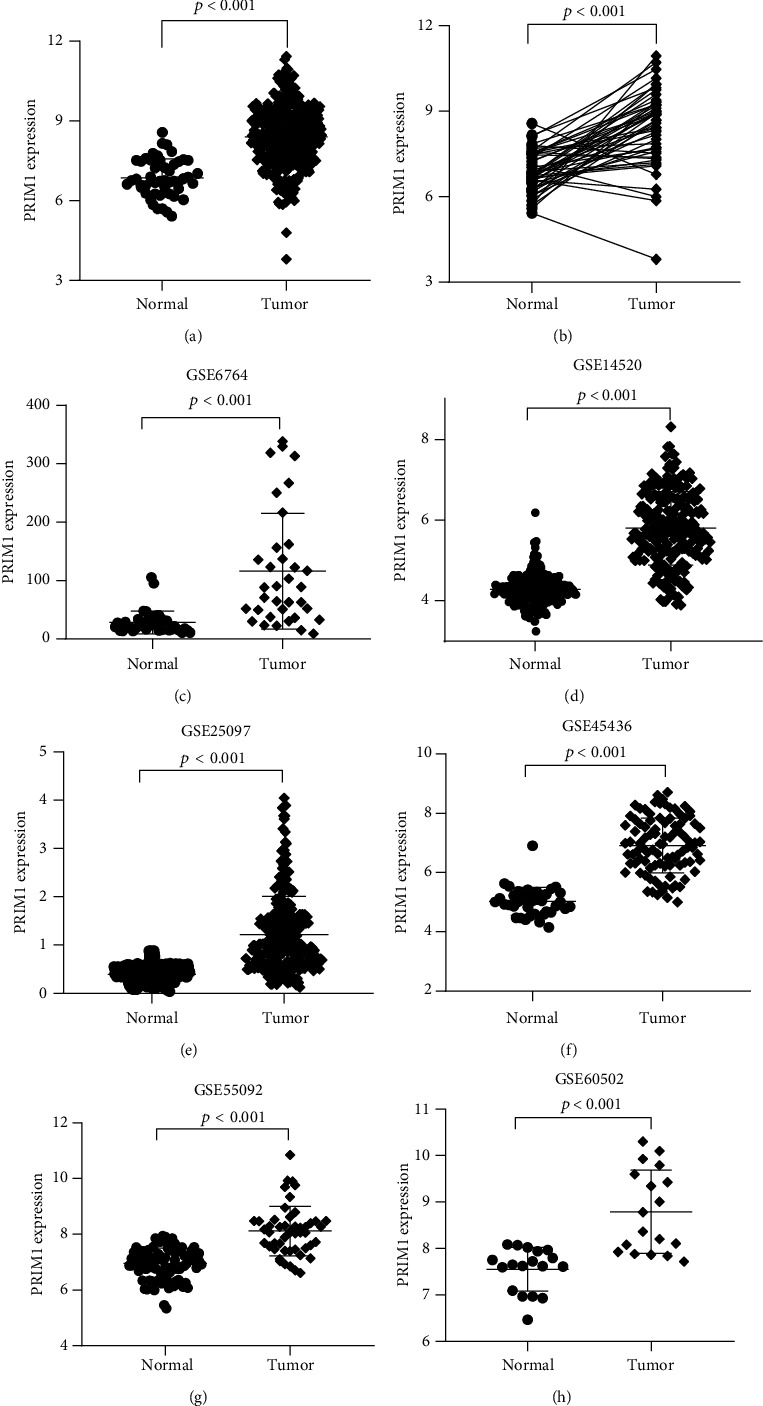
PRIM1 expression was displayed in HCC using data from TCGA and GEO databases. (a) The expression of PRIM1 was evaluated in HCC tissues (*n* = 360) compared with normal tissues (*n* = 49) based on TCGA database. (b) PRIM1 expression was exhibited in HCC tissues (*n* = 49) compared with matched adjacent normal liver tissues (*n* = 49) based on TCGA database. (c–h) PRIM1 expression was evaluated in HCC tissues compared with normal tissues according to the GEO database (GSE25097, GSE6764, GSE14520, GSE45436, GSE55092, and GSE60502). PRIM1: DNA Primase Subunit 1; TCGA: The Cancer Genome Atlas; HCC: hepatocellular carcinoma; GEO: Gene Expression Omnibus.

As presented in Figure 1(a), PRIM1 overexpression was observed in HCC tissues (*n* = 360) based on the gene expression data from TCGA database, compared with that in normal liver tissues (*n* = 49). Moreover, PRIM1 overexpression exhibited in HCC tissues (*n* = 49), compared with that in adjacent liver tissues (*n* = 49) (Figure 1(b)). A higher expression of PRIM1 exhibited in HCC tissues than that in the adjacent ones based on six datasets from the GEO database (GSE25097, GSE6764, GSE14520, GSE45436, GSE55092, and GSE60502) (Figures 1(c)–1(h)).

**Figure 2:**
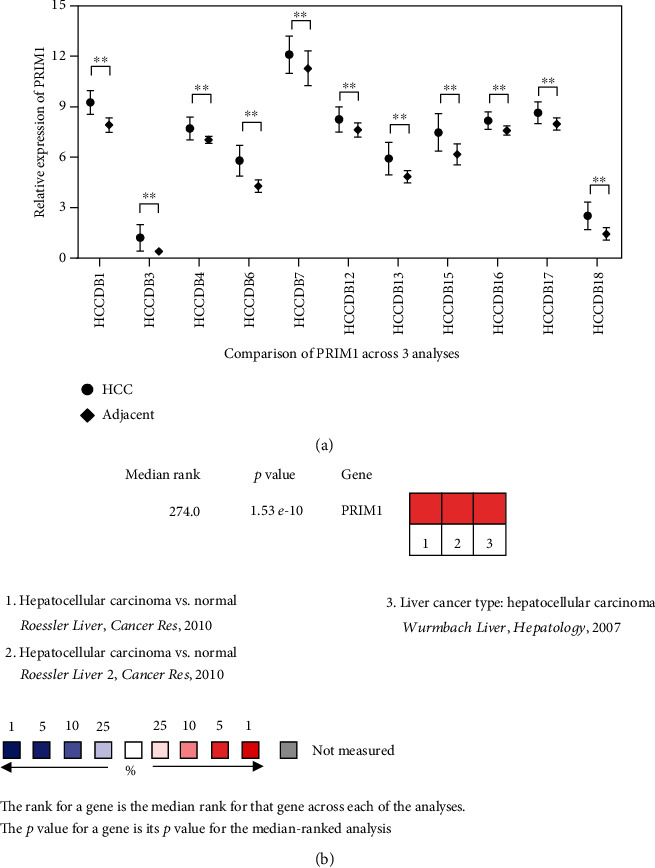
HCCDB and ONCOMINE databases were applied to evaluate the expression of PRIM1 in HCC. (a) The expression of PRIM1 expression was assessed in HCC using the HCCDB database. (b) PRIM1 expression was investigated in HCC using the ONCOMINE database. ^∗^*p* < 0.05, ^∗∗^*p* < 0.01, and ^∗∗∗^*p* < 0.001. PRIM1: DNA Primase Subunit 1; HCC: hepatocellular carcinoma; HCCDB: Integrative Molecular Database of Hepatocellular Carcinoma.

>As revealed by an analysis of the Integrative Molecular Database of Hepatocellular Carcinoma (HCCDB) (Figure 2(a)) and ONCOMINE databases (Figure 2(b)), the PRIM1 expression was remarkably enhanced in HCC tissues.

In a word, these data revealed that the mRNA level of PRIM1 was upregulated in HCC, and these results suggested that enhanced expression of PRIM1 might be closely linked with HCC pathogenesis.

### 3.3. Relationship between the Expression of PRIM1 and Clinicopathological Characteristics in HCC Patients

To further explore the clinical relevance of PRIM1 mRNA expression in HCC, the relationship between PRIM1 expression and the clinicopathological characteristics, such as sex, age, stage, body mass index (BMI), grade, alpha-fetoprotein (AFP) level, platelet, and race, was examined (Table 2). Univariate logistic regression analysis suggested that PRIM1 expression, a categorical dependent variable, was strongly associated with a poor prognosis. A high PRIM1 level in HCC was significantly associated with stage (OR = 1.75 [1.07-2.89] for I-II vs. III-IV; *p* = 0.026), grade (OR = 2.27 [1.47-3.55] for G1-G2 vs. G3-G4; *p* < 0.001), and AFP level (OR = 2.45 [1.37-4.49] for ≤400 ng/ml vs. >400 ng/ml; *p* = 0.003). Additionally, no association between the expression of PRIM1 and other clinicopathological characteristics, such as age (*p* = 0.089), sex (*p* = 0.910), BMI (*p* = 0.053), platelet (*p* = 0.434), and race (*p* = 0.396), was found. These results implied that a more advanced stage, high grade, and high AFP level seemed to be attributable to elevated PRIM1 expression.

### 3.4. The Relationship between PRIM1 Expression and OS in HCC Patients

**Figure 3:**
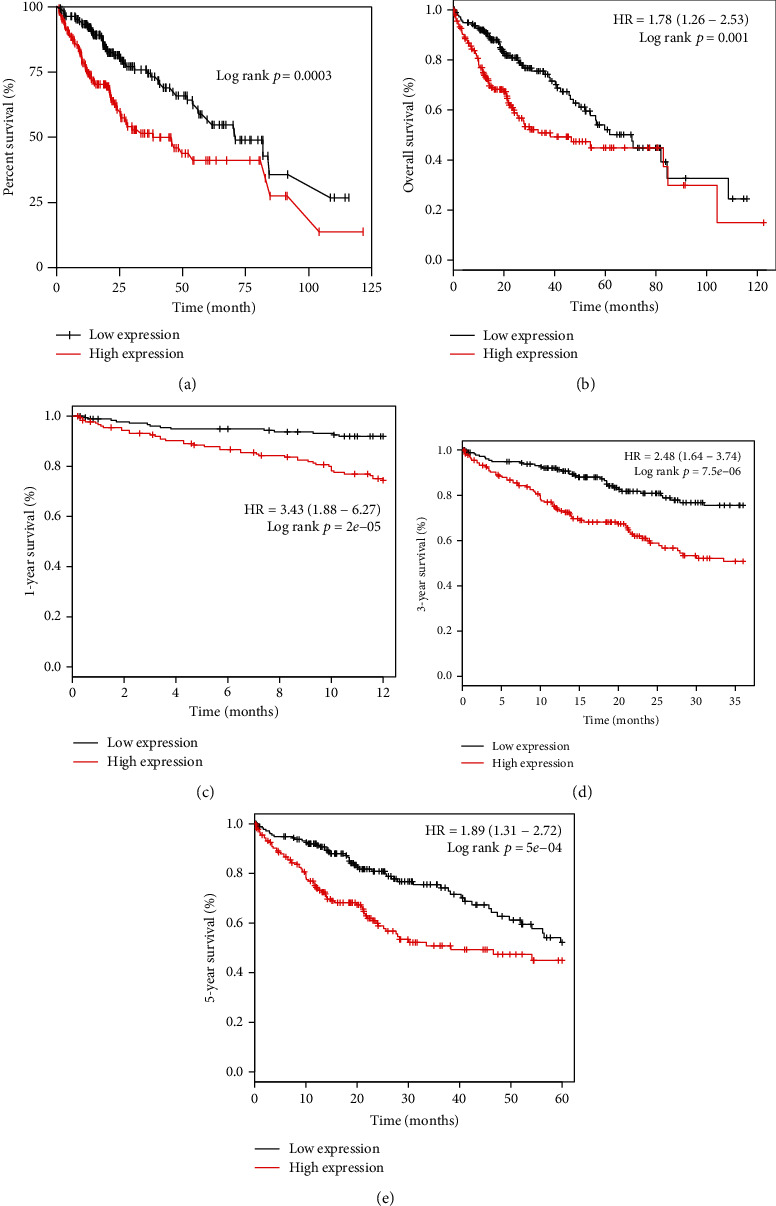
Kaplan-Meier survival analysis of PRIM1 expression in HCC patients. (a) OS time of HCC patients grouped by PRIM1 median expression based on TCGA database. (b) OS time of HCC patients grouped by PRIM1 median expression based on the Kaplan-Meier plotter database. (c) The relationship between PRIM1 median expression and 1-year OS in HCC based on the Kaplan-Meier plotter database. (d) The relationship between PRIM1 median expression and 3-year OS in HCC based on the Kaplan-Meier plotter database. (e) The relationship between PRIM1 median expression and 5-year OS in HCC according to the Kaplan-Meier plotter database. PRIM1: DNA Primase Subunit 1; HCC: hepatocellular carcinoma; TCGA: The Cancer Genome Atlas; OS: overall survival.

As displayed in Figure 3(a), Kaplan-Meier analysis was executed based on the data from TCGA database using survival packages in R, which revealed that the overexpression of PRIM1 had an unfavorable OS in patients with HCC (log-rank *p* < 0.001). As shown in Figure 3(b), subsequent analysis based on the Kaplan-Meier plotter database was consistent with this result. Moreover, subgroup analysis found that PRIM1 overexpression might be considered a risk factor for the 1-year (HR = 3.44 (1.88-6.27), log-rank *p* = 2*e* − 05), 3-year (HR = 2.48 (1.64-3.74), log-rank *p* = 7.5*e* − 06), and 5-year (HR = 1.89 (1.31-2.72), log-rank *p* = 5*e* − 04) OS in patients with HCC (Figures 3(c)–3(e)).

**Figure 4:**
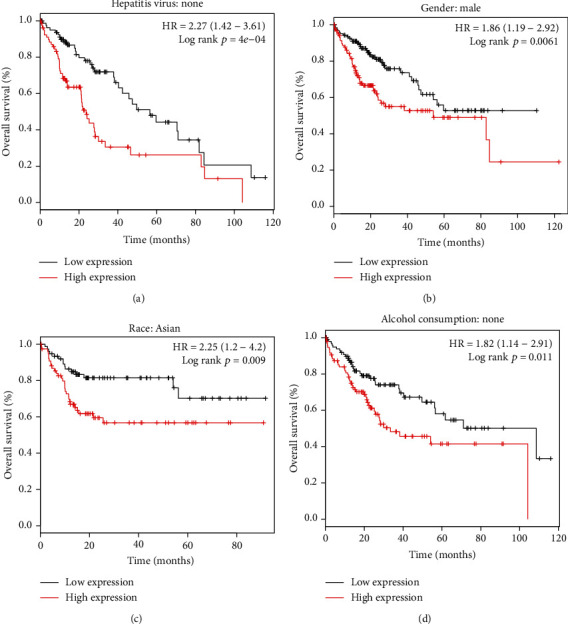
Subgroup analyses of OS in different HCC populations grouped by PRIM1 median expression according to the Kaplan-Meier plotter database. (a) OS of HCC patients without hepatitis virus infection grouped by PRIM1 expression. (b) OS of male HCC patients grouped by PRIM1 expression. (c) OS of Asian HCC patients grouped by PRIM1 expression. (d) OS of HCC patients without alcohol consumption grouped by PRIM1 expression. PRIM1: DNA Primase Subunit 1; HCC: hepatocellular carcinoma; OS: overall survival.

Additionally, subgroup survival analysis was further executed in various patient populations. PRIM1 overexpression had a remarkable correlation with reduced OS in HCC patients without hepatitis virus infection (HR = 2.27 (1.42-3.61), log-rank *p* = 4*e* − 04, Figure 4(a)). Moreover, enhanced PRIM1 expression had a remarkable association with reduced OS in males (HR = 1.86 (1.19-2.92), log-rank *p* = 0.0061, Figure 4(b)). Furthermore, PRIM1 overexpression significantly contributed to the poor OS in Asian HCC (HR = 2.25 (1.2-4.2), log-rank *p* = 0.009, Figure 4(c)). Additionally, PRIM1 expression was a risk factor for OS in HCC patients without alcohol consumption (HR = 1.82 (1.14-2.91), log-rank *p* = 0.011, Figure 4(d)).

**Figure 5:**
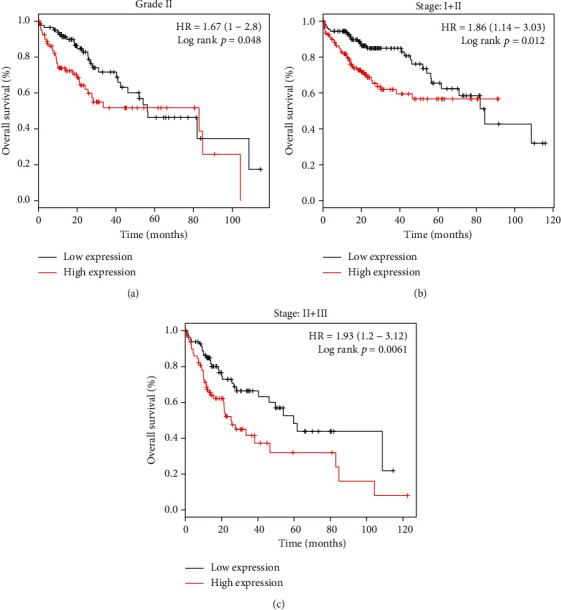
Subgroup analyses of OS in different HCC grades and stages grouped by PRIM1 median expression according to the Kaplan-Meier plotter database. (a) OS of HCC patients with grade II grouped by PRIM1 expression. (b) OS of stage I and II HCC patients grouped by PRIM1 expression. (c) OS of HCC patients with stage II+III grouped by PRIM1 expression. PRIM1: DNA Primase Subunit 1; HCC: hepatocellular carcinoma; OS: overall survival.

A high PRIM1 level significantly contributed to worse OS in HCC patients with grade II (HR = 1.67 (1-2.8), log-rank *p* = 0.048) in Figure 5(a). Additionally, an enhanced expression of PRIM1 had a remarkable association with decreased OS in stage I-II HCC patients (HR = 1.86 (1.14-3.03), log-rank *p* = 0.012, Figure 5(b)) and stage II-III patients (HR = 1.93 (1.2-3.12), log-rank *p* = 0.0061, Figure 5(c)).

### 3.5. PRIM1 Is an Unfavorable Prognostic Factor in HCC

To further explore the prognostic role of PRIM1 in HCC, univariate and multivariate analyses were conducted (Table 3). The univariate analysis showed that elevated PRIM1 expression had a remarkable association with reduced survival (HR: 1.32; 95% CI: 1.06–1.65; *p* = 0.014). Moreover, PRIM1 expression was independently associated with overall survival according to multivariate analysis (HR: 1.31; 95% CI: 1.06–1.62; *p* = 0.027). Additionally, we found that Asian populations had worse survival compared to that in the non-Asian people (*p* < 0.05). Overall, these results indicated that elevated PRIM1 expression might have a crucial role in HCC occurrence and development.

**Figure 6:**
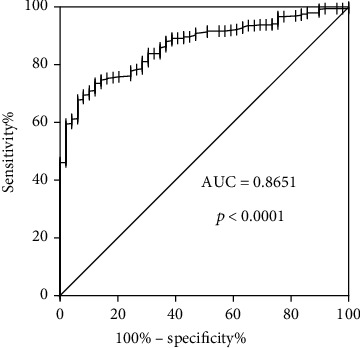
The ROC curve analysis of PRIM1 in HCC. ROC: receiver operating characteristic; PRIM1: DNA Primase Subunit 1; HCC: hepatocellular carcinoma.

To further investigate the potential value of PRIM1 in HCC, the area under the receiver operating characteristic (AUROC) curve for PRIM1 was calculated. As displayed in Figure 6, the value of AUROC reached 0.8651 (*p* < 0.001), and the results implied that PRIM1 might work as a novel and promising molecular biomarker for HCC.

### 3.6. GSEA Identifies PRIM1-Related Signaling Pathways

**Figure 7:**
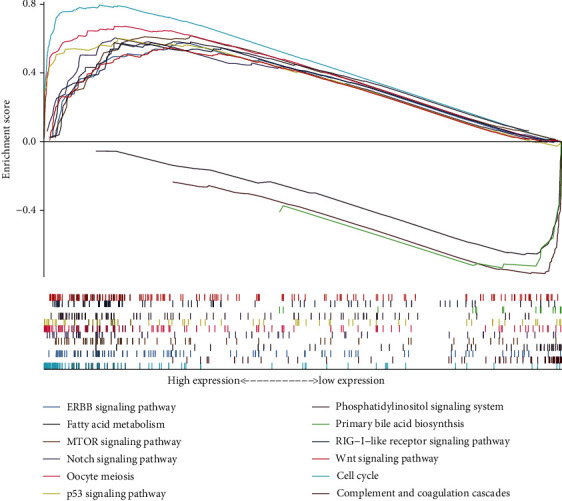
GSEA identifies PRIM1-related oncogenic signaling pathways in HCC. PRIM1: DNA Primase Subunit 1; HCC: hepatocellular carcinoma; GSEA: gene set enrichment analysis.

Gene set enrichment analysis (GSEA) was employed to distinguish significant differences between low and high PRIM1 expression datasets. Twelve signaling pathways were significantly enriched according to the normalized enrichment score (NES) (NOM *p* < 0.05, FDR *q* value < 0.25). As shown in Figure 7 and Table 4, cell cycle, oocyte meiosis, p53 signaling pathway, Wnt signaling pathway, mTOR signaling pathway, ERBB signaling pathway, phosphatidylinositol signaling system, notch signaling pathway, and RIG-I-like receptor signaling pathway were differentially enriched in PRIM1 high expression phenotype, indicating that PRIM1 expression was closely related to cell growth and death, signal transduction, and immune system.

### 3.7. Correlation between Tumor-Infiltrating Immune Cells (TIICs) and PRIM1 Expression in HCC

**Figure 8:**
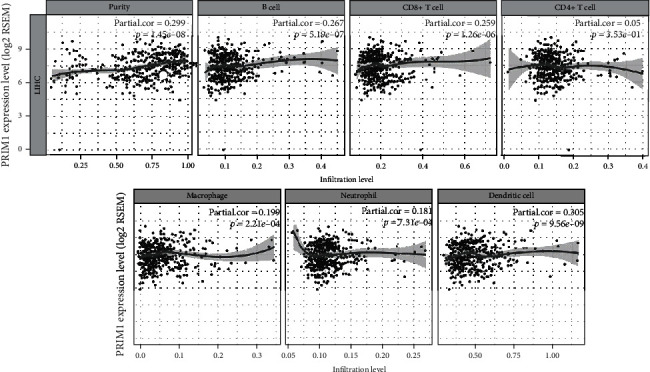
Correlation between TIICs and PRIM1 expression in HCC. PRIM1: DNA Primase Subunit 1; HCC: hepatocellular carcinoma; TIICs: tumor-infiltrating immune cells.

As shown in Figure 8, PRIM1 expression had a positive relationship with the number of B cells (partial correlation, 0.267; *p* = 5.19*e* − 07), CD8+ T cells (partial correlation, 0.259; *p* = 1.26*e* − 06), neutrophils (partial correlation, 0.181; *p* = 7.31*e* − 04), macrophages (partial correlation, 0.199; *p* = 2.21*e* − 04), and dendritic cells (partial correlation, 0.305; *p* = 9.56*e* − 09), indicating that PRIM1 expression might be closely linked with immunotherapy.

## 4. Discussion

In the past few years, although therapies of hepatocellular carcinoma (HCC) had achieved significant progress, HCC remained to have high mortality and morbidity rates due to misdiagnosis or delayed diagnosis [29, 30]. It is imperative to identify underlying biomarkers for HCC. Previous results have shown that a particular relationship appears between gene expression and accurate diagnosis. However, it remains a huge challenge to identify a valid biomarker [31–33]. As far as we know, the potential prognostic value of PRIM1 in HCC is still confused. Moreover, we describe the features of PRIM1 in HCC based on The Cancer Genome Atlas (TCGA) and Gene Expression Omnibus (GEO) databases for the first time, and we centered attention on the underlying impact of PRIM1 on HCC.

High PRIM1 expression was connected with poorly differentiated tumors and poorer survival outcomes in breast cancer [14]. In this study, we initially discovered that PRIM1 expression was significantly increased in HCC using TCGA and GEO databases, which is entirely consistent with the data from the Integrative Molecular Database of Hepatocellular Carcinoma (HCCDB) and ONCOMINE databases. Moreover, we found that HCC patients with high PRIM1 expression showed worse outcomes and lower overall survival. Furthermore, logistic regression showed that PRIM1 expression as a dependent variable was closely related to the poor prognosis. Additionally, multivariate Cox analysis implied that PRIM1 expression was an independent novel prognostic factor for HCC. Collectively, our results indicated that PRIM1 appears to be a desirable prognostic molecular biomarker for HCC. Further studies will be needed to verify the role of PRIM1 in HCC.

Primase Subunit 1 (PRIM1) encodes DNA primase small subunit, which has the catalytic function as enzymes. DNA primase small subunit complexed with polymerase *α* exerted an import part in DNA replication, indicating that the pol*α*-primase complex might be an underlying target [9, 10]. Previous results have shown that it was necessary to change the conformation of the primer for the initiation and elongation of RNA synthesis [14]. PRIM1 catalyzed RNA synthesis, and it was crucial for the accumulation and stimulation of the DNA damage response. Mutations of PRIM1 induced apoptosis through the ATM-Chk2-p53 pathway [14, 34]. Moreover, PRIM1 might have a synthetically lethal relationship with ATR, indicating that targeting the pol*α*-primase complex might be an effective treatment strategy [13]. However, the underlying molecular mechanism of PRIM1 in HCC remains enigmatic.

A preliminary gene set enrichment analysis (GSEA) was employed to identify PRIM1-related oncogenic pathways, indicating that PRIM1 expression was closely related to cell growth and death, signal transduction, and immune system. Furthermore, the investigation on the correlation between tumor-infiltrating immune cells (TIICs) and PRIM1 expression in HCC showed that PRIM1 expression was positively correlated with the number of B cells, neutrophils, macrophages, and dendritic cells, indicating that PRIM1 expression might be a potential biomarker for immunotherapy in HCC. More studies are needed to elucidate its role in HCC.

## 5. Conclusions

Our results indicated that PRIM1 might be a desirable molecular biomarker in HCC. Moreover, PRIM1 might be a conceivable therapeutic target for HCC.

## Figures and Tables

**Table 1:** TCGA hepatocellular carcinoma patient characteristics.

Clinical factors	Total (*n* = 360)	%
Sex		
Male	243	67.5
Female	117	32.5
BMI (kg/m^2^)		
<18.5	21	6.4
18.5~24.99	152	46.5
25-29.99	87	26.6
>30	67	20.5
Stage		
I	167	49.7
II	81	24.1
III	84	25.0
IV	4	1.2
Grade		
G1	53	14.9
G2	171	48.2
G3	120	33.8
G4	11	3.1
Age at diagnosis (y)		
<55	113	30.0
≥55	247	70.0
AFP (ng/ml)		
≤400	207	76.7
>400	63	23.3
Platelet (10^3^/mm^3^)	24.1 (0.004-499)	
Race		
Asian	155	44.3
White	176	50.3
Black or African American	17	4.9
American Indian or Alaska Native	2	0.6

Note: TCGA: The Cancer Genome Atlas; BMI: body mass index; AFP: alpha-fetoprotein.

**Table 2:** The relationship between PRIM1 expression and clinical pathological characteristics (logistic regression).

Clinical characteristics	Total (*N*)	Odds ratio	*p* value
Sex (male vs. female)	360	0.97 (0.63-1.52)	0.910
BMI (<25 kg/m^2^ vs. ≥25 kg/m^2^)	327	0.65 (0.42-1.00)	0.053
Stage (I-II vs. III-IV)	336	1.75 (1.07-2.89)	0.026
Grade (G1-G2 vs. G3-G4)	355	2.27 (1.47-3.55)	<0.001
Age (<55 y vs. ≥55 y)	360	0.68 (0.43-1.06)	0.089
AFP (≤400 ng/ml vs. >400 ng/ml)	270	2.45 (1.37-4.49)	0.003
Platelet (10^3^/mm^3^)	295	1.00 (1.00-1.00)	0.434
Race (Asian vs. non-Asian)	350	0.83 (0.55-1.27)	0.396

Notes: BMI: body mass index; AFP: alpha-fetoprotein; PRIM1: DNA Primase Subunit 1.

**Table 3:** Univariate and multivariate analyses of clinicopathological factors for OS in HCC.

Clinical characteristics	HR	*p* value
Univariate analysis		
Sex (male vs. female)	1.47 (0.90-2.42)	0.126
BMI (<25 kg/m^2^ vs. ≥25 kg/m^2^)	1.24 (0.76-2.03)	0.384
Stage (I-II vs. III-IV)	1.54 (0.88-2.68)	0.131
Grade (G1-G2 vs. G3-G4)	1.39 (0.85-2.27)	0.191
Age (<55 y vs. ≥55 y)	1.38 (0.80-2.36)	0.247
AFP (≤400 ng/ml vs. >400 ng/ml)	1.02 (0.58-1.79)	0.938
Platelet (10^3^/mm^3^)	1.00 (1.00-1.00)	0.377
Race (Asian vs. non-Asian)	2.22 (1.30-3.77)	0.003
PRIM1 expression (high vs. low)	1.32 (1.06-1.65)	0.014
Multivariate analysis		
Race (Asian vs. non-Asian)	2.21 (1.30-3.76)	0.006
PRIM1 expression (high vs. low)	1.31 (1.06-1.62)	0.027

Notes: BMI: body mass index; AFP: alpha-fetoprotein; PRIM1: DNA Primase Subunit 1.

**Table 4:** Pathways were enriched in the PRIM1 expression differential phenotype.

Gene set name	NES	NOM *p* val	FDR *q* val
Cell cycle	2.289	<0.001	<0.001
Oocyte meiosis	2.182	<0.001	<0.001
p53 signaling pathway	1.987	<0.001	0.005
Wnt signaling pathway	1.837	0.004	0.018
MTOR signaling pathway	1.832	<0.001	0.017
ERBB signaling pathway	1.825	0.006	0.017
Phosphatidylinositol signaling system	1.769	0.004	0.020
Notch signaling pathway	1.761	0.008	0.021
RIG-I-like receptor signaling pathway	1.790	0.002	0.019
Complement and coagulation cascades	-2.164	<0.001	<0.001
Primary bile acid biosynthesis	-1.711	0.019	0.177
Fatty acid metabolism	-1.687	0.027	0.149

Notes: PRIM1: DNA Primase Subunit 1; ES: enrichment score; NES: normalized ES; NOM *p* val: normalized *p* value.

## Data Availability

Previously reported gene expression data (GSE25097, GSE6764, GSE14520, GSE45436, GSE55092, and GSE60502) were applied to support this study and are available at the GEO database (https://www.ncbi.nlm.nih.gov/geo). TCGA data were obtained from https://portal.gdc.cancer.gov/.
